# Cyclosporin A in Membrane Lipids Environment: Implications for Antimalarial Activity of the Drug—The Langmuir Monolayer Studies

**DOI:** 10.1007/s00232-015-9814-9

**Published:** 2015-06-16

**Authors:** Patrycja Dynarowicz-Łątka, Anita Wnętrzak, Katarzyna Makyła-Juzak

**Affiliations:** Department of General Chemistry, Faculty of Chemistry, Jagiellonian University, Ingardena 3, 30-060 Kraków, Poland; Institute of Physics, Jagiellonian University, Łojasiewicza 11, 30-348 Kraków, Poland

**Keywords:** Cyclosporin A, Interactions, Membrane lipids, Langmuir monolayers, Model of normal and parasitized erythrocyte membrane

## Abstract

Cyclosporin A (CsA), a hydrophobic cyclic peptide produced by the fungus *Tolypocladium inflatum*, is well known for its high efficiency as an immunosuppressor for transplanted organs and anti-inflammatory properties; however, it is also active as antiparasitic (antimalarial) drug. Antimalarial mechanism of CsA action lacks a detailed understanding at molecular level. Due to a high lipophilicity of CsA, it is able to interact with lipids of cellular membrane; however, molecular targets of this drug are still unknown. To get a deeper insight into the mode of antimalarial activity of CsA, it is of utmost importance to examine its interactions with membrane components. To reach this goal, the Langmuir monolayer technique, which serves as a very useful, easy to handle and controllable model of biomembranes, has been employed. In this work, the interactions between CsA and main membrane lipids, i.e., cholesterol (Chol), 2-oleoyl-1-palmitoyl-3-phosphocholine (POPC), and sphingomyelin (SM), have been investigated. Attractive interactions are observed only for CsA mixtures with SM, while repulsive forces occur in systems containing remaining membrane lipids. Taking into consideration mutual interactions between membrane lipids (Chol–SM; Chol–POPC and SM–POPC), the behavior of CsA in model erythrocyte membrane of normal and infected cells has been analyzed. Our results prove strong affinity of CsA to SM in membrane environment. Since normal and parasitized erythrocytes differ significantly in the level of SM, this phospholipid may be considered as a molecular target for antimalarial activity of CsA.

**Electronic supplementary material:**

The online version of this article (doi:10.1007/s00232-015-9814-9) contains supplementary material, which is available to authorized users.

## Introduction

**Fig. 1:**
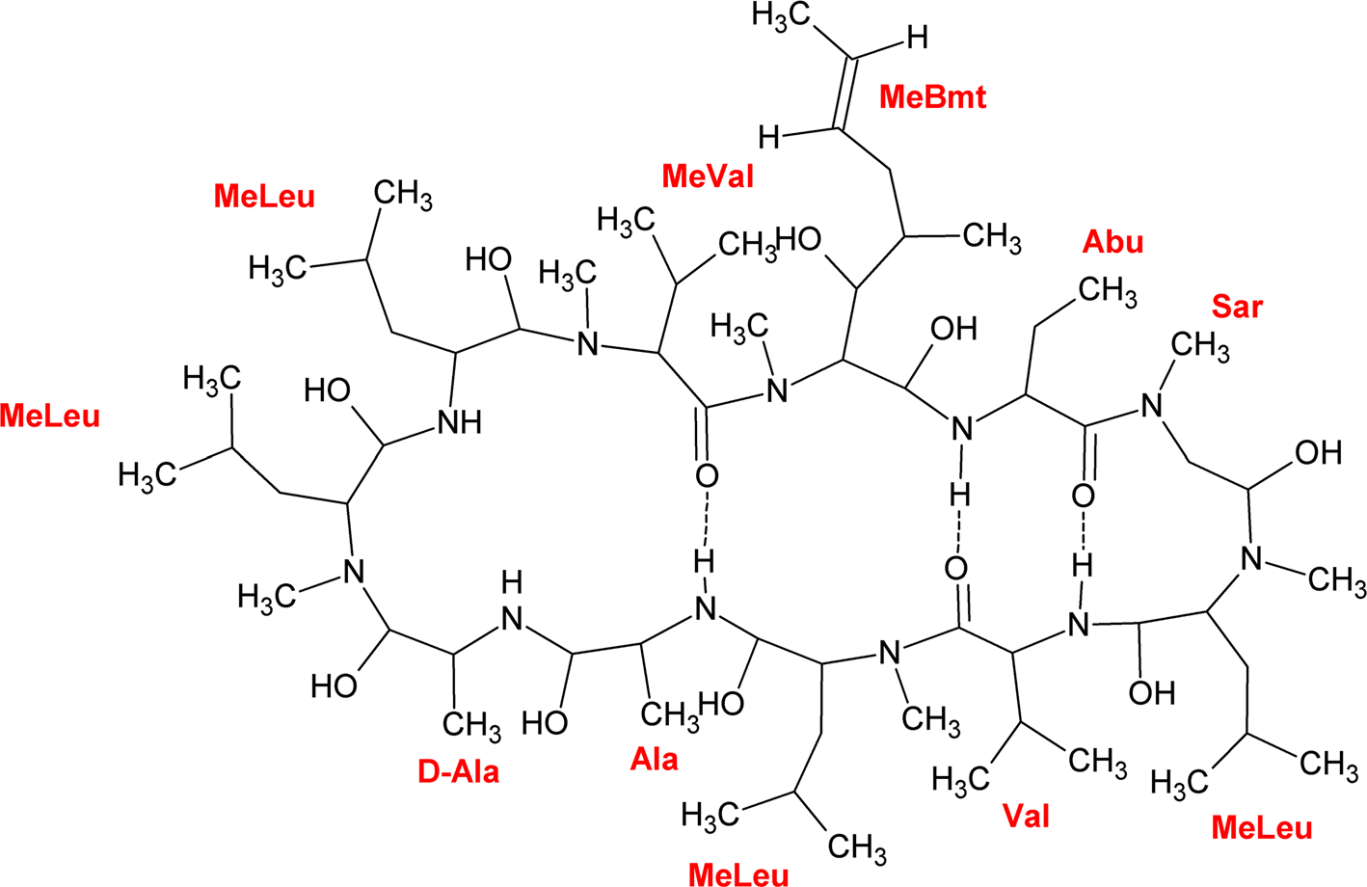
Structure of Cyclosporin A (according to Wenger et al. 1994) with the standard numbering of the amino acid residues

Cyclosporin A (CsA) is a hydrophobic cyclic peptide produced by soil fungus *Tolypocladium inflatum* and composed of 11 amino acids, mostly methylated, that are linked by several hydrogen bonds (Fig. 1) (Borel et al. 1979; Czogalla 2009). It is known for preventing the rejection of transplanted organs. Apart from its immunosuppressive properties, other important biological properties have been reported for CsA, including anti-inflammatory, antifungal, antiviral (anti-HIV), and antiparasitic (antimalarial) action (see Kallen et al. 1997 for a review). Although the mechanism of immunosuppressive activity has been well recognized as being due to CsA association with cyclophilins—specific proteins of immune cells (Kallen et al. 1997), its other pharmacological activities are not well understood. Of a special interest is antiparasitic (antimalarial) action of CsA, the mechanism of which remains unclear up to now (Bell et al. 1996). Malaria—a mosquito-transmitted human disease, known from ancient times, still remains a major health concern, mainly in tropical countries, leading to millions death each year. It is caused by unicellular protozoan parasites of the *Plasmodium* genus (*P. falciparum* and *P. vivax* being the major species involved in human malaria) and is transmitted to humans by infected *Anopheles* mosquitos bites (Aditya et al. 2013). Following the period of growth and multiplication in host liver cells, the parasites are released into circulation and invade erythrocytes by endocytosis. Inside red blood cell, the parasite surrounds with vacuolar membrane (PVM) to ensure its survival (Aditya et al. 2013).

Parasitized erythrocytes undergo substantial modifications in structure (shape deformation (Holz 1977)) and function—most important alterations include modified transport (Cabantchik 1989), lower exchange rate of cholesterol as well as marked increase in membrane permeability, and fluidity (Hsiao et al. 1991; Maguire and Sherman 1990 and references therein). These changes occur in consequence of a different composition of infected red blood cells (IRBC) as compared to normal ones (NRBC) as reported in many papers (specified in Maguire and Sherman 1990 for infections with *Plasmodium falciparum*). To summarize, main differences in erythrocyte membrane composition upon infection with malarial parasite involve changes in lipid and fatty acid composition (Maguire and Sherman 1990; Shalmiev and Ginsburg 1993). Firstly, although the cholesterol content in IRBC is increased, the cholesterol-to-phospholipids ratio (Chol/PL) is ca. half as smaller as in NRBC. Secondly, IRBC contain less sphingomyelin (SM) (a 37–47 % decrease *versus* NRBC). Another important difference concerns fatty acids composition—generally, the amount of polyunsaturated fatty acids in normal erythrocyte phospholipids was found to be much higher than in infected cells (Hsiao et al. 1991). It is worthy pointing out here that the membrane composition of IRBC is complicated as it reflects, in fact, total composition of multimembrane system, including plasma membrane of the host and parasite, the PVM as well as nucleus membrane of *Plasmodium* (Sherman 1979). Nevertheless, these results obviously indicate that significant changes of membrane organization of the host erythrocytes occur as a result of parasitization.

This paper is aimed at verifying whether the IRBC membrane is a pharmacologic target of CsA. As mentioned above, the mode of antimalarial activity of CsA has not been elucidated so far although it is known to be different to that for quinoline-containing drugs, used, alternatively to CsA, in classical antimalarial treatment. Namely, the mode of action of quinoline antimalarials is based on their accumulation in parasites vacuole and inhibition of the polymerization of heme (produced by parasites from hemoglobin digestion), which is toxic to the parasites in the free form (Sherman 1979). CsA does not interfere with heme polymerization (Azouzi et al. 2011), and therefore, its antimalarial activity must be based on a different mechanism. Many studies using liposomes as membrane models (reviewed in Czogalla 2009) have proved that CsA incorporates and interacts with lipid membranes. Most interesting finding is that CsA penetrates into lipid membranes, having preferences for fluid/gel boundaries, and perturbs acyl chains especially near the head group (Lambras and Rahman 2004; Soderlund et al. 1999). Interestingly, cholesterol was found to decrease penetration of CsA into DPPC bilayer in a pressure-dependent way (Soderlund et al. 1999). These results unambiguously prove membrane activity of CsA and suggest that modified upon infection, erythrocyte membrane may be a key factor for the antiparasitic property of this peptide. There are examples in literature showing that changes in membrane organization, associated to pathogenic processes, can significantly alter the drug-membrane affinity. Such a behavior was observed for new generation antitumor drugs of phospholipids-like structure (e.g. edelfosine) (Dynarowicz-Łątka and Hąc-Wydro 2014) for which tumor cell membrane, markedly differing in lipid composition *versus* normal cell membrane, was a selective target for their antineoplastic activity. Other examples are polyene antibiotics, such as amphotericin B (Seoane et al. 1999; Gruszecki et al. 2003; Foglia et al. 2014) or antimicrobial lipopeptide: mycosubtilin (Nasir and Besson 2012), the antimycotic activity of which is related to their affinities to ergosterol-containing membranes, typical for fungi.

To find out how different lipid composition modulates the interaction of CsA, we have modeled erythrocyte membrane of normal and infected cells and studied the effect exerted by the addition of CsA using the Langmuir monolayer technique (Gaines 1966). This method serves as a very useful, easy to handle, and controllable model of biomembranes (Brockman 1999). Cyclosporin A, although slightly soluble in water (Czogalla 2009), was found to form insoluble monolayers at the water/air interface (Wieldmann and Jordan 1991). It has already been studied in mixed Langmuir monolayers, however, only in 2-component systems with different phospholipids (DPPC, DPPE, DPPS, DPPG) (Fahr and Reiter 1999; Sandez et al. 1999; Sandez Macho et al. 2001). The obtained results indicated immiscibility and lack of interactions between the peptide and PL. Due to its limited solubility in water, CsA was subjected to penetration experiments from bulk water into Langmuir monolayers from different membrane lipids—DPPC, cholesterol (Söderlund et al. 1998; Azouzi et al. 2010), mixed DPPC/cholesterol (Söderlund et al. 1998), and sphingomyelin (SM) (Azouzi et al. 2010) to verify whether it can act on lipid target. Penetration experiments revealed that cyclosporin A inserts into all the studied lipids, however, with a preference to sphingomyelin (Azouzi et al. 2010). Cholesterol, on the other hand, was found to hinder the penetration of CsA into PC monolayer (Söderlund et al. 1998).

In this work, we have employed Langmuir monolayer technique for systematic studies of the interactions between CsA and erythrocyte membrane to better understand the antiparasitic activity of this drug.

## Experimental

### Materials

The following compounds were purchased and used: cyclosporin A (Cell Signaling Technology), cholesterol (Sigma-Aldrich), POPC (2-oleoyl-1-palmitoyl-3-phosphocholine), and sphingomyelin (egg chicken) (both from Avanti Polar Lipids). All these products were of high purity (>99 %) and were used as received. The investigated compounds were dissolved in a spectroscopic grade chloroform/methanol (4:1 v/v) mixture (Sigma-Aldrich, p.a.) with typical concentration of 0.2–0.5 mg/mL. Mixed solutions were obtained by mixing proper volumes of respective stock solutions. Models of erythrocyte membrane of healthy and infected cells were prepared by mixing cholesterol (Chol) with most abundant phospholipids (PL) of RBC, i.e., POPC and SM. It is well known that in NRBC, the Chol:PL ratio is 0.9 (Yawata 2003); however, upon infection with malarial parasite it is ca. half less (Maguire and Sherman 1990). Within phospholipids, the SM:PC ratio is 0.5 in normal cells [Table 2, Shalmiev and Ginsburg 1993)], while in infected cells, it is decreased. Following data, presented in (Shalmiev and Ginsburg 1993, Table 1) for SM:PC ratio for erythrocytes infected with various strains of *P. falciparum*, is 0.2 on average.

**Table 1:** Parameters of geometrical molecular packing; hydrophobic chain length; and hydrophobic volume were calculated by formulas $V = \left( {27.4 + 26.90n_{\text{c}} } \right)({\AA}^{3} )$, $l_{\text{c}} = \left( {1.5 + 1.265n_{\text{c}} } \right)({\AA})$ (according to Israelachvili 2011)

Compound	*a* (Å^2^)	*l*_c_ (Å)	*V* (Å^3^)	*s*	Geometrical shape
Cholesterol	19	17.25	400	1.22	Inverted truncated cone
SM	71.7	20.475	837.6	0.57	Truncated cone
POPC	71.7	20.475	910	0.62	Truncated cone

### Methods

The *π*/*A* isotherms were recorded using KSV Langmuir trough of the total area = 760 cm^2^ while films visualization was performed with Brewster angle microscopy (ultra-BAM, Accurion GmbH, Goettingen, Germany), applying the methodology described elsewhere (Dynarowicz-Łątka et al. 2013).

## Results and Discussion

In the first step of our work, pure CsA was thoroughly investigated in Langmuir monolayers. Former papers proved film-forming abilities of this peptide (Wieldmann and Jordan 1991; Fahr and Reiter 1999; Sandez et al. 1999; Sandez Macho et al. 2001; Azouzi et al. 2010). However, one must be aware of the fact that the results of monolayer experiments, especially for peptides, may vary, depending on the experimental conditions applied. Indeed, in literature, there were some discrepancies as regards the limiting area. Namely, the value of 230–260 Å^2^/molecule was obtained from the isotherms by Fahr and Reiter 1999. Slightly larger areas (of about 280 Å^2^) were reported in Refs. (Sandez et al. 1999; Sandez Macho et al. 2001; Azouzi et al. 2010). However, much smaller value (of 130 Å^2^) was found in Ref. (Wieldmann and Jordan 1991). These differences in limiting areas were suggested as being due to a very low speed of compression applied in the latter paper, which may result in either lost of monolayer material from the interface to bulk phase or conformational changes of peptide molecules (Fahr and Reiter 1999). Also a possibility of the peptide adsorption to the Teflon barriers of the trough (Fahr and Reiter 1999) was implied as a potential source of errors resulting in the observed different lift-off area values. To clarify these issues, we have performed a number of systematic studies on the influence of various experimental conditions on the characteristic of the *π*/*A* isotherm from cyclosporin A and employed Brewster angle microscope (BAM) to visualize the structure of the studied monolayers. In a set of preliminary experiments, we have found out that neither the initial surface density of molecules deposited on the free water surface nor change in compression speed within the range of 5–30 mm/min modifies the course of the isotherm in a significant way (see Fig. S1a, b, Supplementary materials). In our experiments, applying a standard rate of compression of 20 mm/min, the limiting area (obtained by extrapolation at *π* = 0) equals 260 Å^2^. To get insight into physical state of CsA monolayer, the compression moduli (*C*_S_^−1^ = −*A* (d*π/*d*A*)) values have been calculated from the isotherms’ datapoints and plotted as a function of mean molecular area (Fig. S1a, inset). According to *C*_S_^−1^ values (Davies and Rideal 1963), CsA forms a liquid-type monolayer (maximum *C*_S_^−1^ is attained slightly above 100 mN/m). This occurs at the mean molecular area of 230 Å^2^, which corresponds exactly to the theoretical cross-section area of CsA (Fahr and Reiter 1999). Under the routine compression speed of 20 mm/min, the texture of monolayers is completely homogeneous within surface pressures 0–20 mN/m (Fig. S1b, image 1), and only in the vicinity of film collapse, bright crystallites appear (image 2), which grow in number upon further compression (image 3), which explains non-entirely horizontal post-collapse segment. For the applied compression speed ranging from 5 to 30 mm/min, the monolayer collapse (determined as the intersection point of two straight lines fitted to the experimental points below and above divergence of the *π*/*A* isotherm) occurs at 27 mN/m. However, when the monolayer was compressed with a very low speed (1 mm/min) so that the entire film compression on the applied trough took ca. 145 min, the crystallites started to appear already at 5 mN/m (Fig. S1b, image 4) and—in consequence—the film collapsed at markedly lower surface pressure. It is just evident that the collapse of CsA monolayer occurs via nucleation mechanism. No changes were observed upon changing the barrier material from Teflon to Delrin (S1c). CsA was found to form stable monolayers at low surface pressures (see the results for stability experiments at *π* = 5 and 15 mN/m, Fig. S1d, Supplementary materials). Although these experiments prove that CsA is capable of forming quite stable Langmuir monolayers, one has to be aware of potential errors, resulting from using spreading solvent of greater density than water and Wilhelmy plate made of filter paper (for details see Brzozowska and Figaszewski 2002a) or different initial concentration of spreading solution.

**Fig. 2:**
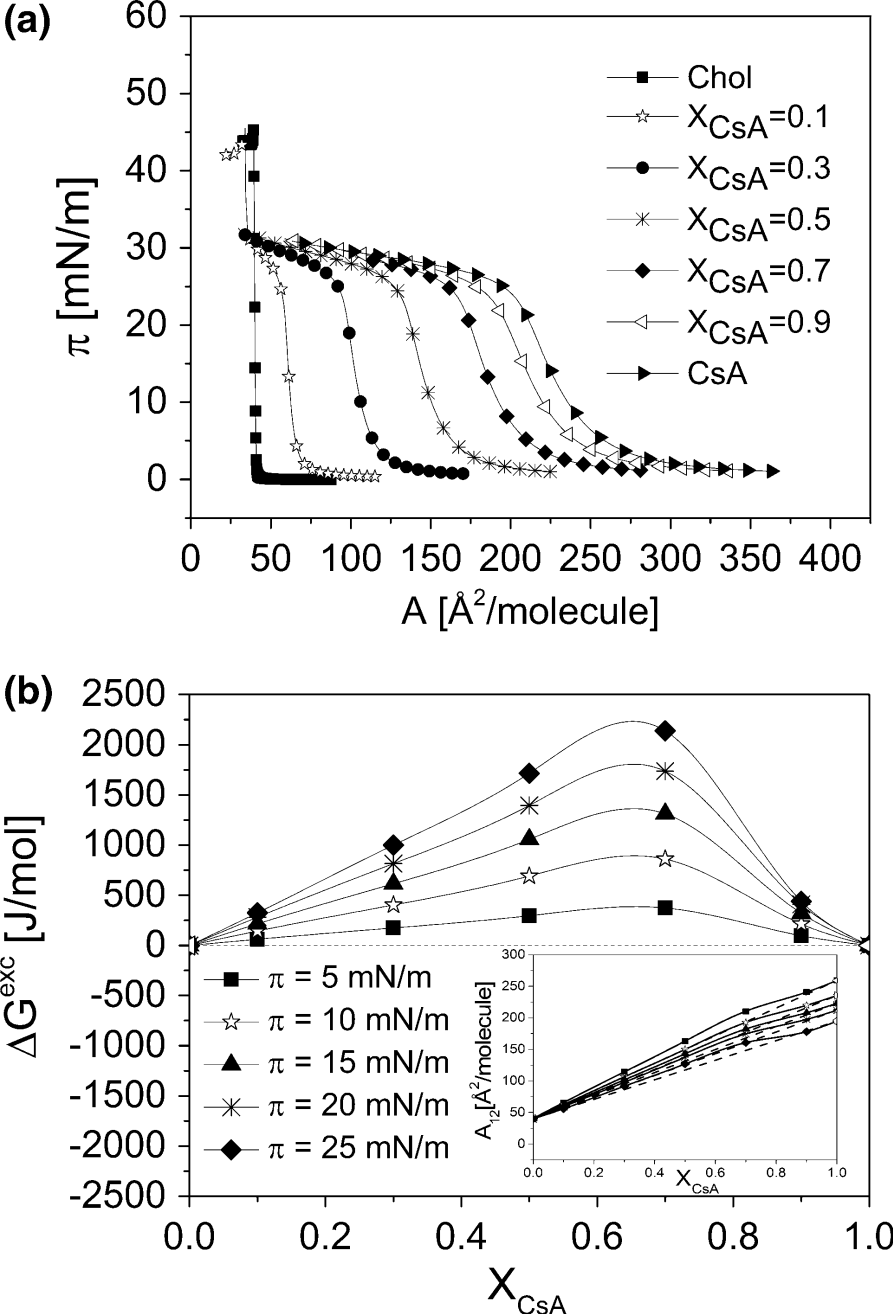
Surface pressure (*π*)–area (*A*) isotherms for CsA, Chol, and their mixtures (**a**); and excess free energy of mixing (Δ*G* ^exc^) versus mixed film composition (*X*_CsA_) plots (**b**). *Inset* mean molecular area (*A*_12_)–*X*_CsA_ plots

**Fig. 3:**
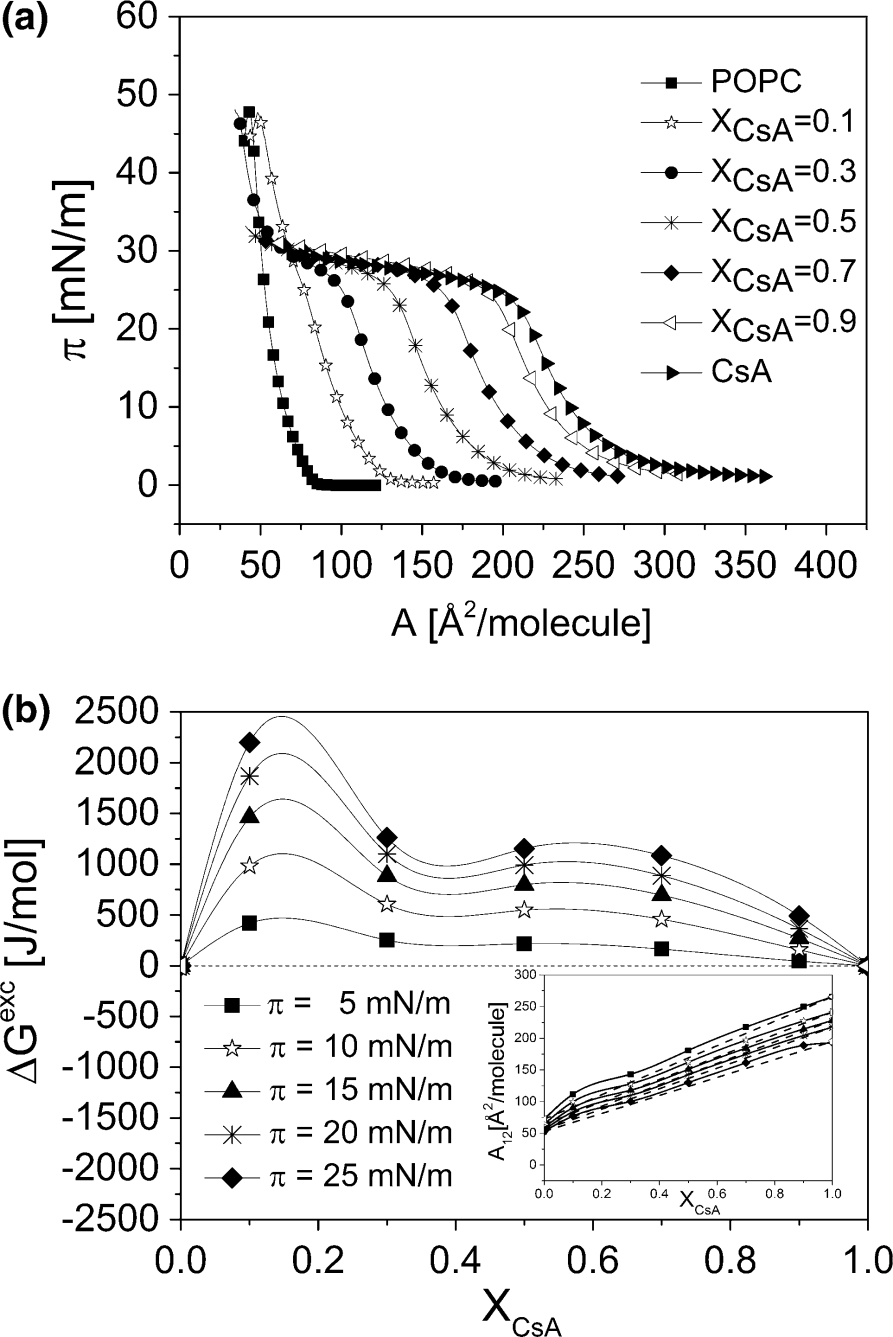
Surface pressure (*π*)–area (*A*) isotherms for CsA, POPC, and their mixtures (**a**); and excess free energy of mixing (Δ*G* ^exc^) versus mixed film composition (*X*_CsA_) plots (**b**). *Inset* mean molecular area (*A*_12_)–*X*_CsA_ plots

**Fig. 4:**
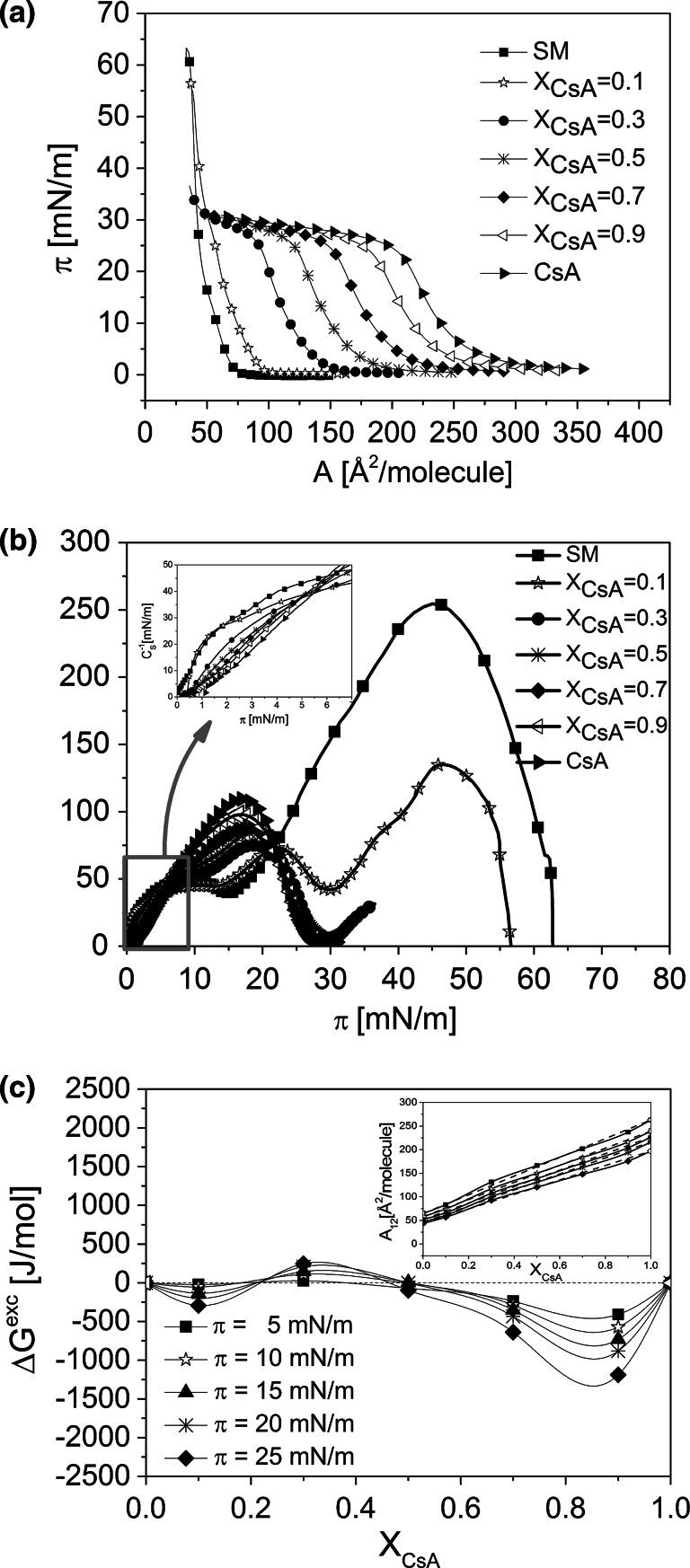
Surface pressure (*π*)–area (A) isotherms for CsA, SM, and their mixtures (**a**); compression modulus (*C*_s_^−1^)–surface pressure (*π*) dependence (**b**) and excess free energy of mixing (Δ*G* ^exc^) versus mixed film composition (*X*_CsA_) plots (**c**). *Inset* mean molecular area (*A*_12_)–*X*_CsA_ plots

To be able to interpret the results of CsA in model erythrocyte membrane, it is first necessary to examine interactions between the drug and main constituent membrane lipids (Chol, POPC, and SM). Cholesterol is known for its crucial role in regulating membrane physicochemical properties in eukaryotic cell (Crane and Tamm 2004) as well as its involvement—together with sphingomyelin—in the formation of ordered lipid rafts (Fan et al. 2010), which have been hypothesized to be a site of action of some drugs (e.g., antitumor ether phospholipids (Heczkova and Slotte 2006). Phosphatidylcholines (PC), on the other hand, are most abundant phospholipids building biological membranes. In particular, in erythrocyte membrane, they constitute ca. 40 % of all phospholipids (Shalmiev and Ginsburg 1993). It is also necessary to take into consideration mutual interactions between membrane lipids: PC/Chol, SM/Chol, and SM/PC, which are analyzed below.

As proved in many papers (reviewed in: Maget-Dana 1999; Deleu et al. 2014; Dynarowicz-Łątka and Hąc-Wydro 2014; Stefaniu et al. 2014), using the Langmuir monolayer technique, one can easily model cell membranes by mixing membrane lipids in an appropriate proportion, and then monitor changes in membrane properties caused by the addition of a drug of interest. This has been done in the second part of this study, where interactions between CsA and 3-component model membrane system of normal and infected red blood cells have been investigated.

### Isotherms of CsA Mixed with Membrane Lipids

Since CsA and all the studied membrane lipids (Chol, POPC, and SM) were proved to form stable Langmuir monolayers on aqueous subphases, the Langmuir monolayer technique can well be applied for studying their mutual interactions.

Mixed monolayers were prepared for five different mole fractions of CsA (*X*_CsA_): 0.1, 0.3, 0.5, 0.7, and 0.9. The obtained isotherms are presented in panels a, Figs. 2, 3, and 4. Although the texture of monolayers of  pure lipids can be found elsewhere, however, to facilitate the readers analysis of the images for more complicated systems, they are provided in Fig. S2 Supplementary materials.

In all the studied systems, mixed monolayers lie between those for pure components. The isotherms recorded for Chol, POPC, and SM are in good agreement with those published elsewhere (see for example Cadena-Nava et al. 2006 for cholesterol, Yun et al. 2003 for POPC and Prenner et al. 2007 for SM). A characteristic feature of SM monolayer is the presence of a phase transition from the liquid-expanded to liquid-condensed state (Smaby et al. 1994; Prenner et al. 2007), which is visible both in the course of the isotherms and also on the *C*_S_^−1^ versus *π* plots as a broad plateau, which spans from 6 to 15 mN/m (Fig. 4b). As seen in Figs. 2, 3, and 4, monolayers of the investigated membrane lipids collapse at significantly higher surface pressures (*π*_coll_ = 44, 50 and 69 mN/m for Chol, POPC, and SM, respectively) as compared to CsA (*π*_coll_ = 27 mN/m). For mixed monolayers rich in membrane lipid, two collapse pressures are seen in the course of the isotherms—one corresponds exactly to the *π*_coll_ for pure CsA, and the other one, at higher pressure, to a particular lipid. In general, the presence of two independent collapses in the course of an isotherm from two film-forming molecules, corresponding to collapse pressure values for pure components, evidences for their immiscibility in a monolayer (Gaines 1966). The first collapse corresponds to the ejection of a substance collapsing at lower pressures, while the other one—to the expulsion of the second substance, collapsing at higher pressures. Thus, for the discussed mixtures, it is evident that at a lower collapse pressure, CsA is first removed from the monolayer, and afterwards the remaining component (lipid). Similar behavior of two collapses can be expected for other mixed film compositions; however, the second collapse is not visible as it is expected to occur at a low area (due to small proportion of lipid in the mixed monolayer), which is out of the moving barrier range.

It is evident that at surface pressures above the first collapse, all the studied systems are immiscible. However, at lower surface pressures, the components can mix and interact. To get insight into the behavior of CsA/membrane lipids mixtures at pressures below the first collapse, qualitative (mean molecular area, *A*_12_) and quantitative (excess free enthalpy changes, Δ*G*^exc^) parameters of interaction have been calculated (Gaines 1966).

For two-component systems, the mean area per molecule, *A*_12_ is defined as follows *A*_12_ = *A*_1_*X*_1_ + *A*_1_*X*_1_, wherein *A*_1_, *A*_2_ are the molecular area of single component at the same surface pressure and *X*_1_, *X*_2_ are the mole fractions of components 1 and 2 in the binary film. Δ*G*^exc^ is defined as $\int\limits_{0}^{\pi } {A^{\text{exc}} {\text{d}}\pi }$ and wherein *N* is the Avogadro’s number, and *A*^exc^ = *A*_12_ − *X*_1_*A*_1_ − *X*_2_*A*_2_.

The calculated parameters of interactions, both qualitative (*A*_12_) and quantitative (Δ*G*^exc^) (Figs. 2b, 3b, 4b, together with insets), prove that in mixed monolayers of CsA with membrane lipids, attractive interactions occur only with SM (negative deviations from ideality observed for the majority of mixed films compositions), while repulsive forces occur both in its mixtures with Chol and POPC. Interactions between CsA and SM are therefore of a particular importance, especially regarding the antimalarial activity of this peptide, since this particular sphingolipid is an important component of RBC membrane and—as indicated in the “Introduction” section—its content changes significantly upon infection with *Plasmodium* parasites.

To deeply investigate the effect of CsA on sphingomyelin monolayer, the compression moduli values have been calculated and plotted as a function of surface pressure (Fig. 4c). As it has already been mentioned, CsA forms liquid-type monolayers; however, monolayer of SM gives liquid-condensed type of film. Interestingly, the effect of CsA on SM monolayer is different at surface pressures below and above the phase transition of SM monolayer as compared to higher pressures. As it is evident from the inset of Fig. 4b, at low surface pressures (up to 6 mN/m), the addition of CsA exerts little fluidization on SM monolayer; however, at pressures corresponding to the phase transition and above, the effect of the peptide is opposite. It can be speculated that due to the increase of molecular packing upon compression, SM molecules adopt gradually more vertical orientation, and the attractive van der Waals forces between SM chains and CsA (which is a hydrophobic molecule) increase, making possible the incorporation of the peptide into SM monolayer, which reflects in different pattern of interaction.

The strength of SM-CsA interactions is found to depend on mixed film composition: attractive forces are observed in CsA-rich films, while for SM-rich monolayers, the interactions are very weak and oscillate around zero (Fig. 4b).

### Mutual Interactions Between Membrane Lipids

**Fig. 5:**
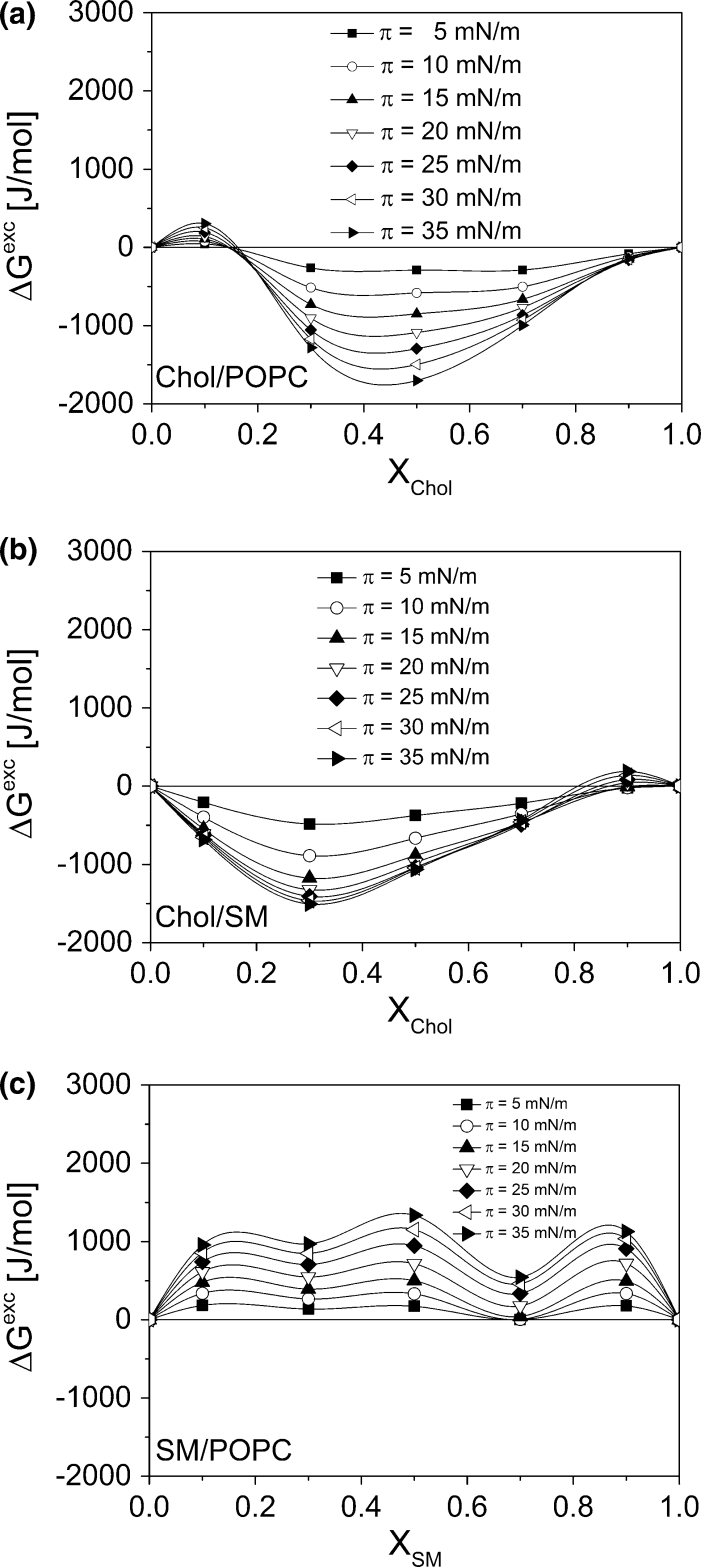
Excess free energy of mixing (Δ*G* ^exc^) versus mixed film composition (*X*) plots for **a** Chol/POPC **b** Chol/SM and **c** SM/POPC systems

Prior to analyze the interactions of CsA with model RBC membrane, which is mimicked as a ternary system of Chol, POPC, and SM, binary mixtures of these membrane lipids, i.e., Chol–POPC; Chol–SM, and POPC-SM, are first discussed. Since the pressure/area curves for the above systems have already been investigated (Wydro et al. 2011; Hąc-Wydro and Dynarowicz-Łątka 2008; Prenner et al. 2007, respectively), herein, we present only the results of interactions (Fig. 5), while the experimental isotherms can be viewed in Supplementary materials, S3. Deviation for linearity in the *A*_12_ versus *X*_lipid_ plots proves that in all the studied system the components mix and interact within the whole composition range. In both systems containing cholesterol, significant attractive forces resulting in condensation of phospholipid monolayers occur. In fact, the condensing effect of sterols (cholesterol being studied most frequently) on phospholipids monolayers has well been known (Smaby et al. 1994). The majority of authors attribute this effect to the formation of stable complexes between a phospholipid and cholesterol molecules. Particularly for phosphatidylcholine-cholesterol mixtures, the formation of complexes has ambiguously been proved (Petelska and Figaszewski, 1998; Brzozowska and Figaszewski 2002a, b). Depending on the kind of a particular PC and physical state of its monolayers, formation of complexes of different stiochiometry (3:1; 2:1 or 1:1) was reported (see for example Dynarowicz-Łątka and Hąc, 2004, and discussion in Dynarowicz-Łątka et al. 2002). For the investigated herein system of Chol–POPC, in the whole range of surface pressures, the stabilization of mixed films of all compositions was reflected by their negative Δ*G*^exc^ values. The particular stability of the 1:1 POPC-cholesterol monolayer, having the most negative Δ*G*^exc^ values (Fig. 5a), suggests that this very composition must be of a particularly favorable arrangement of both components, leading to the formation of surface complexes of 1:1 stoichiometry. These results are in agreement with those already published for POPC–Chol monolayers (Jurak 2013; Wydro et al. 2011; Petelska and Figaszewski 1998; Brzozowska and Figaszewski 2002a).

Cholesterol and another investigated here phospholipid: sphingomyelin are known to exhibit high affinity to each other (Shaikh et al. 2001; Smaby et al. 1994). Chol–SM mixtures mimic lipid rafts (Radhakrishnan et al. 2001; Ohvo-Rekilä et al. 2002), which are known to consist mainly of these two classes of lipids (Simons and Ikonen 1997). High stability of Chol–SM complexes has been proved to be formed in monolayers at the air/water interface (Petelska and Figaszewski 2013). The minimum visible in the Δ*G*^exc^ = *f*(*X*_Chol_) plots (Fig. 5b) corresponds to the mixed film composition of the strongest interactions that occur for Chol–SM films of 1:2 proportion. In the literature, this very composition is attributed to the simplest model lipid raft system (Jablin et al. 2010; Hąc-Wydro et al. 2011) although other authors, e.g., (Thakur et al. 2011), mimic lipid rafts as more complex ternary (POPC + Chol + SM) or quaternary (POPC + Chol + SM + GM1) mixtures of different proportions of its components.

In contrast to cholesterol-containing mixtures, the system of two phospholipids (POPC-SM) shows completely different behavior, i.e., repulsive interactions between both phospholipids, which reflect in positive Δ*G*^exc^ values (Fig. 5c). Such a behavior, leading to the possibility of phase separation, has been observed for mixtures of various phospholipids (Shaikh et al. 2001; Więcek et al. 2008; Dynarowicz-Łątka et al. 2013) as well as for the investigated here mixture of PC-SM (Wydro 2012).

**Fig. 6:**
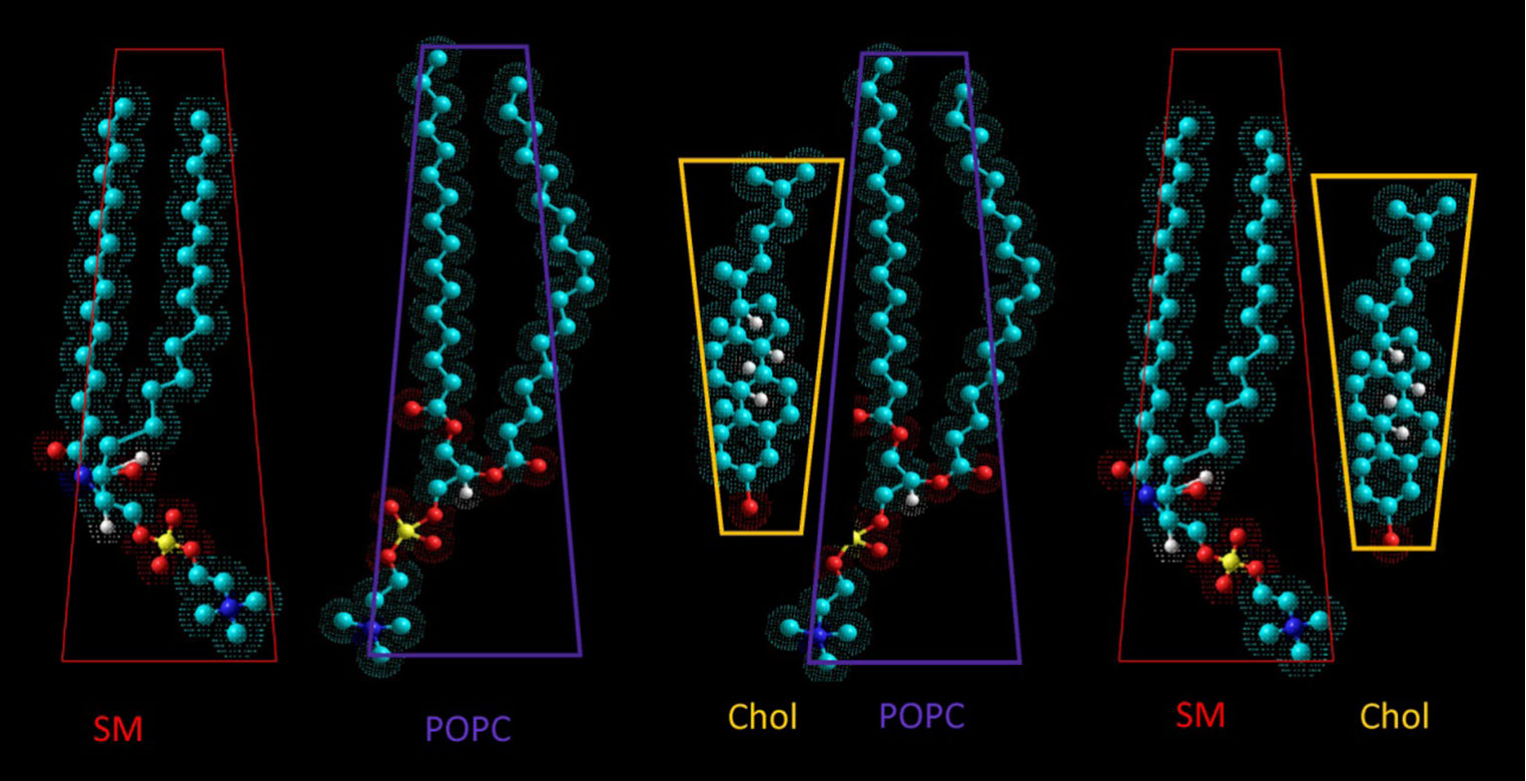
Molecular arrangement between cholesterol, sphingomyelin, and POPC modeled with HyperChem (HyperChem 8.0 2011)

Different affinity of interacting molecules discussed herein (Chol–POPC; Chol–SM versus POPC-SM) can be also analyzed with geometric packing of molecules, which is expressed in terms of a dimensionless critical packing parameter *s* (defined as $s = \frac{V}{{a \cdot l_{\text{c}} }}$) (Israelachvili 2011; Israelachvili et al. 1980) that depends on the head group area *a*, volume *V*, and critical length *l*_c_ of the hydrocarbon chain. Calculated values of parameter *s* (Table 1) reveal inverted truncated cone shape of cholesterol and truncated conical shape of SM and POPC. The thermodynamic analysis, proving the existence of strong attractive interactions between cholesterol and phospholipids, agrees well with the analysis of the geometry of interacting molecules. Namely, cholesterol and phospholipids are of opposite geometry, which ensure their favorable packing (Fig. 6). On the other hand, in the case of two phospholipids, shape complementarity is not optimal, and such an arrangement may lead to phase separation.

### CsA in Model Erythrocyte Membrane

Knowing the interactions between the peptide and membrane lipids as well as mutual interactions between particular membrane lipids, we could proceed and examine the behavior of CsA in more advanced systems, i.e., in model normal and infected erythrocyte membrane, which is composed of three main lipids: phosphatidylcholines, cholesterol, and sphingomyelin mixed in a proper proportion, according to (Yawata 2003).

From the analysis of 2-component mixtures of membrane lipids, it can be expected that in ternary mixtures of Chol–POPC-SM, both phospholipids—due to their mutual repulsive interactions—will be available in their free, unbound form to interact with cholesterol, which will compete to interact with both of them. Taking into consideration that the strength of interactions occurring in Chol–POPC and Chol–SM systems is similar (Fig. 5a vs b), the erythrocyte membrane can be considered as being composed of Chol–SM and Chol–POPC complexes surrounded by free cholesterol molecules.

Normal erythrocyte membrane was modeled as follows: Chol/PL = 0.9 (Hąc-Wydro et al. 2009; Hąc-Wydro 2012), where PL composition (SM/POPC) was equal to 0.5, according to the literature cited already in the “Introduction” section. On the other hand, model of the parasitized membrane was taken as Chol/PL = 0.45 (PL composition: SM/POPC = 0.2).

**Fig. 7:**
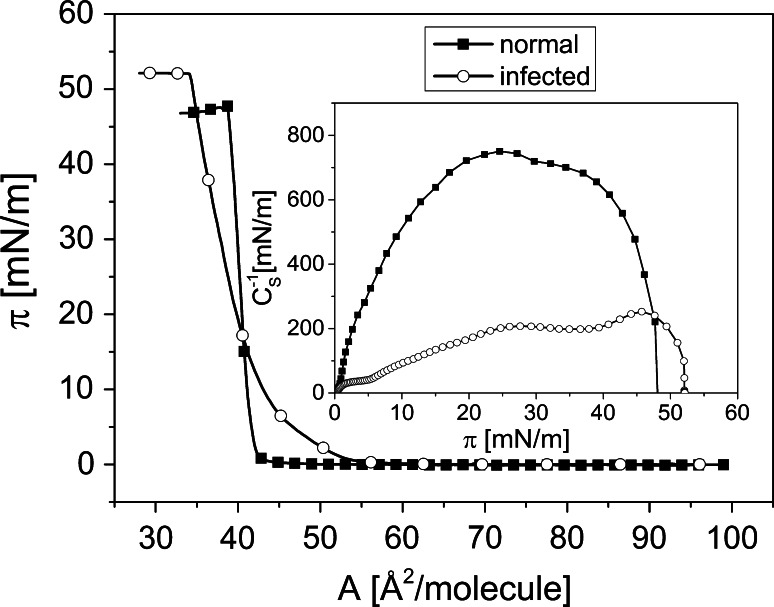
Surface pressure (π)–area (A) isotherms for model normal and infected membrane together with compression modulus (*C*_s_^−1^)–surface pressure (*π*) dependence (*inset*)

The pressure/area isotherms for both model membranes are shown in Fig. 7. At the first glance, one can notice a significant difference in the physical state of model normal versus infected erythrocyte membrane. The isotherm of the latter is more inclined (i.e., the state of the monolayer is more fluid) as compared to vertical isotherm for uninfected membrane, proving its high rigidity. These differences can clearly be seen in *C*_S_^−1^ values, which are shown in the inset of Fig. 7 at the surface pressure of 25 mN/m. More fluid membranes of IRBC versus NRBC were also observed for in vivo studies (Taraschi et al. 1986), and are due to a noticeable lower proportion of Chol/PL in parasitized cells.

**Fig. 8:**
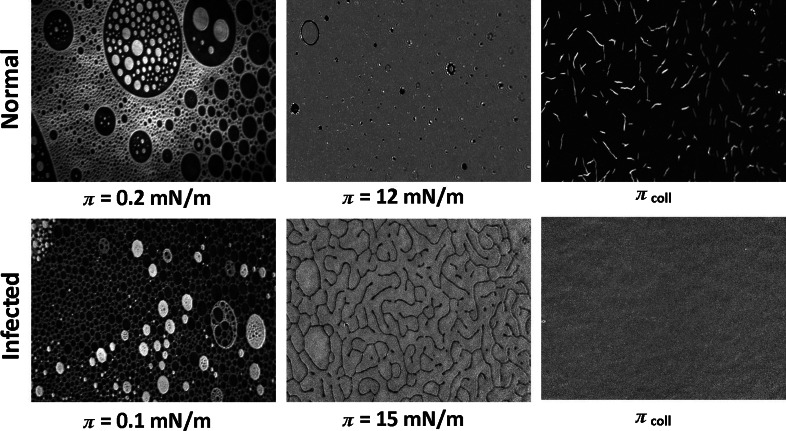
BAM images for model membrane of normal and infected erythrocytes

Significant differences in structure of both membranes are evidenced with BAM images (Fig. 8). The texture of NRBC membrane is almost identical as the monolayer of pure cholesterol (see Fig. S2 for comparison), which can be expected due to the overwhelming proportion of cholesterol in non-infected erythrocytes membrane. On the other side, monolayer mimicking parasitized cells reflects, at low surface pressure, the coexistence of gaseous structures of all three component lipids. Also it is seen that monolayer modeling infected membrane is less condensed as it contains proportionally more expanded phase (the darker the structure, the more expanded it is).

**Fig. 9:**
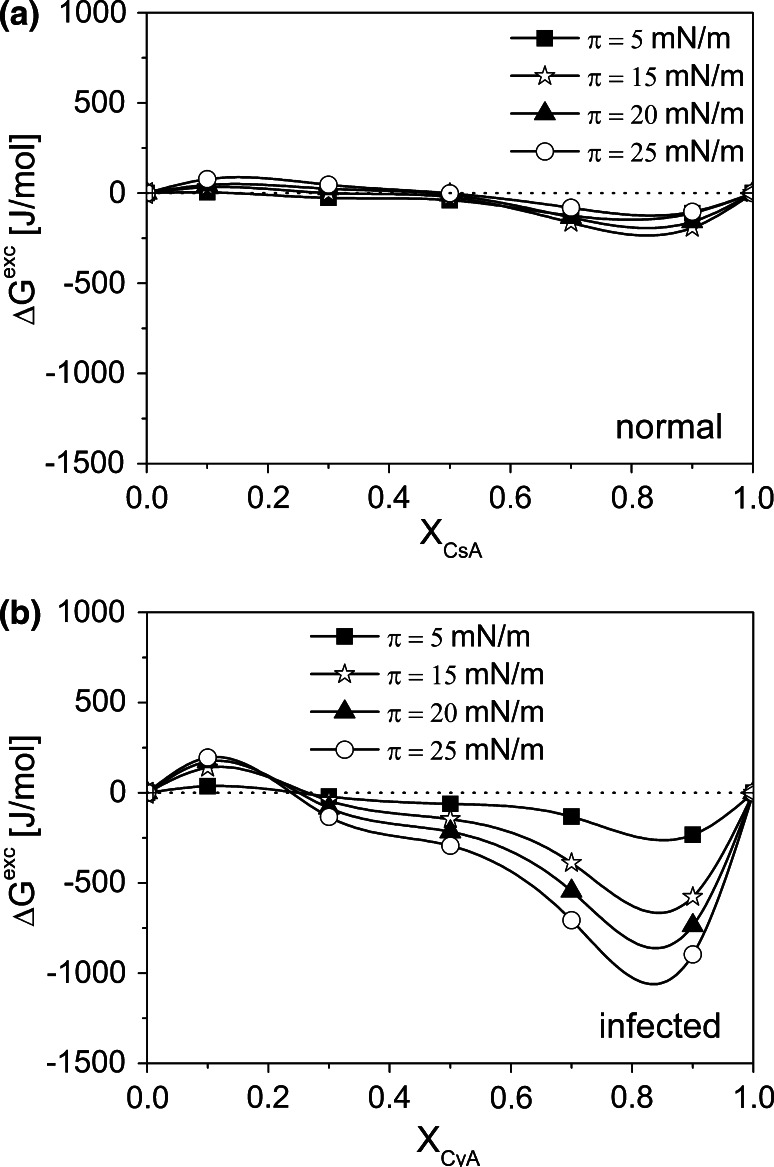
Excess free energy of mixing (Δ*G* ^exc^) versus mixed film composition (*X*_CsA_) plots for normal (**a**) and infected (**b**) model membrane

Due to significant difference in fluidity and texture of NRBC versus IRBC, it may be expected that the introduction of CsA will affect both membranes in quite a different way. Since the pressure/area isotherms solely are not too much informative and at first glance they look similar (S4, Supplementary materials), the parameters of interaction have been calculated for both model systems. For quaternary films, the mean area per molecule *A*_1234_ was obtained directly from the experimental curves *π*–*A* and compared with the values resulting from the additivity rule $A_{1234}^{\text{id}} = \sum\limits_{i = 1}^{4} {A_{i} X_{i} }$. Values of the excess area of mixing were obtained from the following equation: $A^{\text{exc}} = A_{1234} - A_{1234}^{\text{id}}$. Next, the excess free enthalpy changes $\Delta G^{\text{exc}} = N_{A} \int\limits_{0}^{\pi } {A^{\text{exc}} {\text{d}}\pi }$ were calculated and presented in Fig. 9a, b. As can be seen, values of Δ*G*^exc^ substantially differ for both membranes.

Let us analyze CsA in normal erythrocyte membrane. In this model, CsA is embedded in the environment of free cholesterol molecules coexisting with small amounts of SM–Chol and POPC–Chol complexes. As evidenced in “Isotherms of CsA mixed with membrane lipids” section, CsA interacts favorably only with SM, and therefore, in model membrane, CsA will compete with Chol to interact with SM molecules. However, the number of SM molecules available to interact with the peptide is negligible, and as a result, Δ*G*^exc^ values oscillate around zero (Fig. 9a). However, in the infected erythrocyte membrane, the number of SM molecules capable of interacting with CsA is significantly larger. As shown in “Mutual interactions between membrane lipids” section, cholesterol forms more stable complexes with POPC than with SM (lower Δ*G*^exc^ values for Chol–POPC vs Chol–SM). Thus, Chol–SM complexes are more labile and SM molecules can preferentially interact with the peptide. In fact Δ*G*^exc^ values for CsA in infected membrane (Fig. 9b) resemble almost identically the results for CsA–SM mixtures (Fig. 4b).

## Conclusion

Our results prove that the interactions between CsA and erythrocyte membrane are totally dominated by interactions with SM. Different amount of SM molecules available for interacting with the peptide in normal versus infected membrane determines the resultant effect, which is quite different for both studied membrane models. These results may suggest that sphingomyelin can be considered as a molecular target attracting CsA to the Plasmodium-infected erythrocytes.

## Electronic supplementary material

Supplementary material 1 (PDF 938 kb)

Supplementary material 2 (PDF 524 kb)

Supplementary material 3 (PDF 476 kb)

Supplementary material 4 (PDF 369 kb)
