# Supplementary material for: Cyclosporin A in Membrane Lipids Environment: Implications for Antimalarial Activity of the Drug—The Langmuir Monolayer Studies
Source: J Membr Biol. 2015 Jun 16;248(6):1021–32. doi: 10.1007/s00232-015-9814-9 (PMC4611017; doi:10.1007/s00232-015-9814-9)
Supplement: Supplementary file 1 — Supplementary material 1 (PDF 938 kb) [file 232_2015_9814_MOESM1_ESM.pdf]

# Supplementary Material 1

The Journal of Membrane Biology

Cyclosporin A in Membrane Lipids Environment – Implications for Antimalarial Activity of the Drug. The Langmuir Monolayer Studies

Patrycja Dynarowicz-Łątka*, Anita Wnętrzak, Katarzyna Makyła-Juzak

*Corresponding author: ucdynaro@cyf-kr.edu.pl

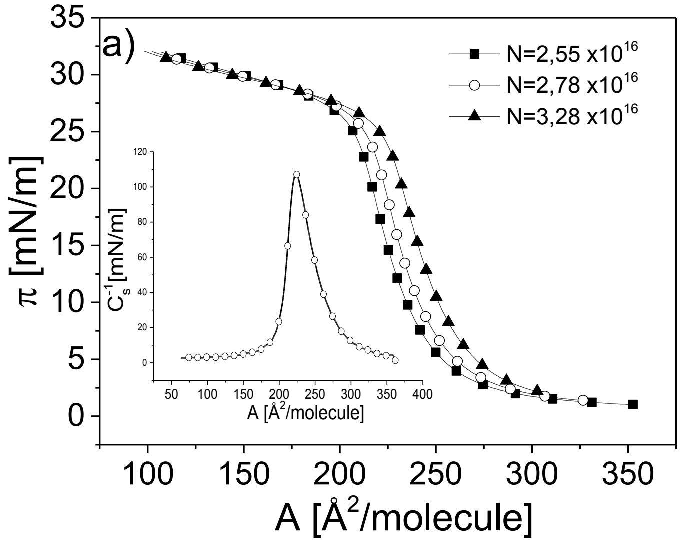


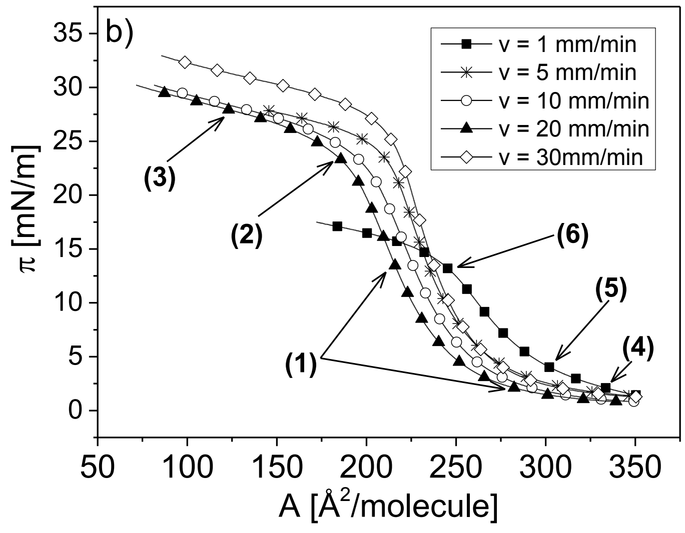

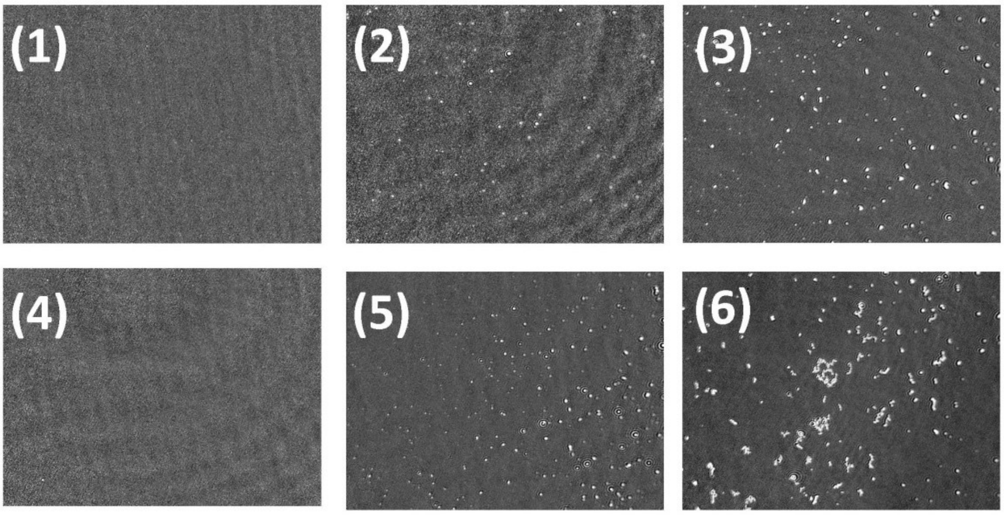
(1)
(2)
(3)
(4)
(5)
(6)

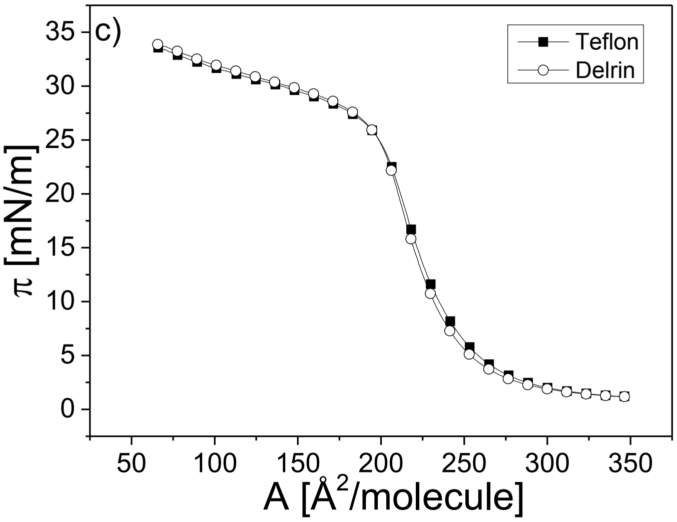
c)
Teflon
Delrin
π [mN/m]
A [Å$^2$/molecule]

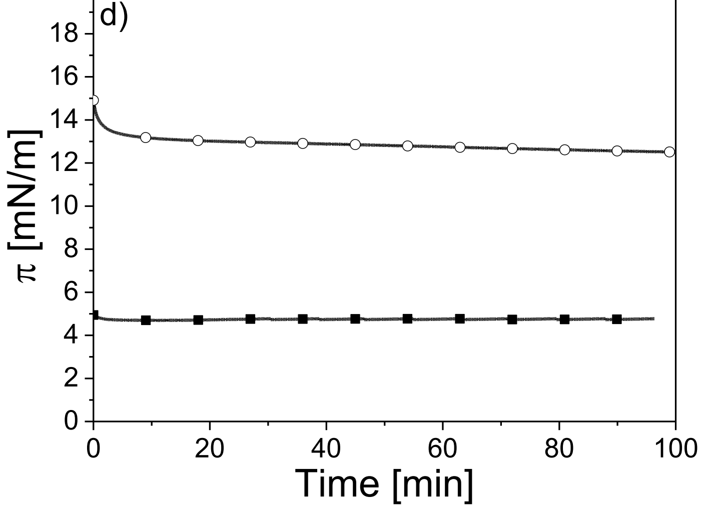
d)
π [mN/m]
Time [min]

**Fig. S1** The influence of experimental conditions on the π-area isotherms of CsA

(a) number of molecules (compression speed 20 mm/min)

(b) compression speed, complemented with BAM images taken at surface pressures indicated by arrows (number of molecules spread at the surface $N=2.55\ x10^{16}$)

c) type of barrier material (compression speed 20 mm/min, number of molecules spread at the surface $N=2.55\ x10^{16}$) , and

(d) static stability experiments of cyclosporin A monolayer.
