# Supplementary material for: Cyclosporin A in Membrane Lipids Environment: Implications for Antimalarial Activity of the Drug—The Langmuir Monolayer Studies
Source: J Membr Biol. 2015 Jun 16;248(6):1021–32. doi: 10.1007/s00232-015-9814-9 (PMC4611017; doi:10.1007/s00232-015-9814-9)
Supplement: Supplementary file 2 — Supplementary material 2 (PDF 524 kb) [file 232_2015_9814_MOESM2_ESM.pdf]

Supplementary Material 2

The Journal of Membrane Biology

Cyclosporin A in Membrane Lipids Environment – Implications for Antimalarial Activity of the Drug. The Langmuir Monolayer Studies

Patrycja Dynarowicz-Łątka*, Anita Wnętrzak, Katarzyna Makyła-Juzak

*Corresponding author: ucdynaro@cyf-kr.edu.pl

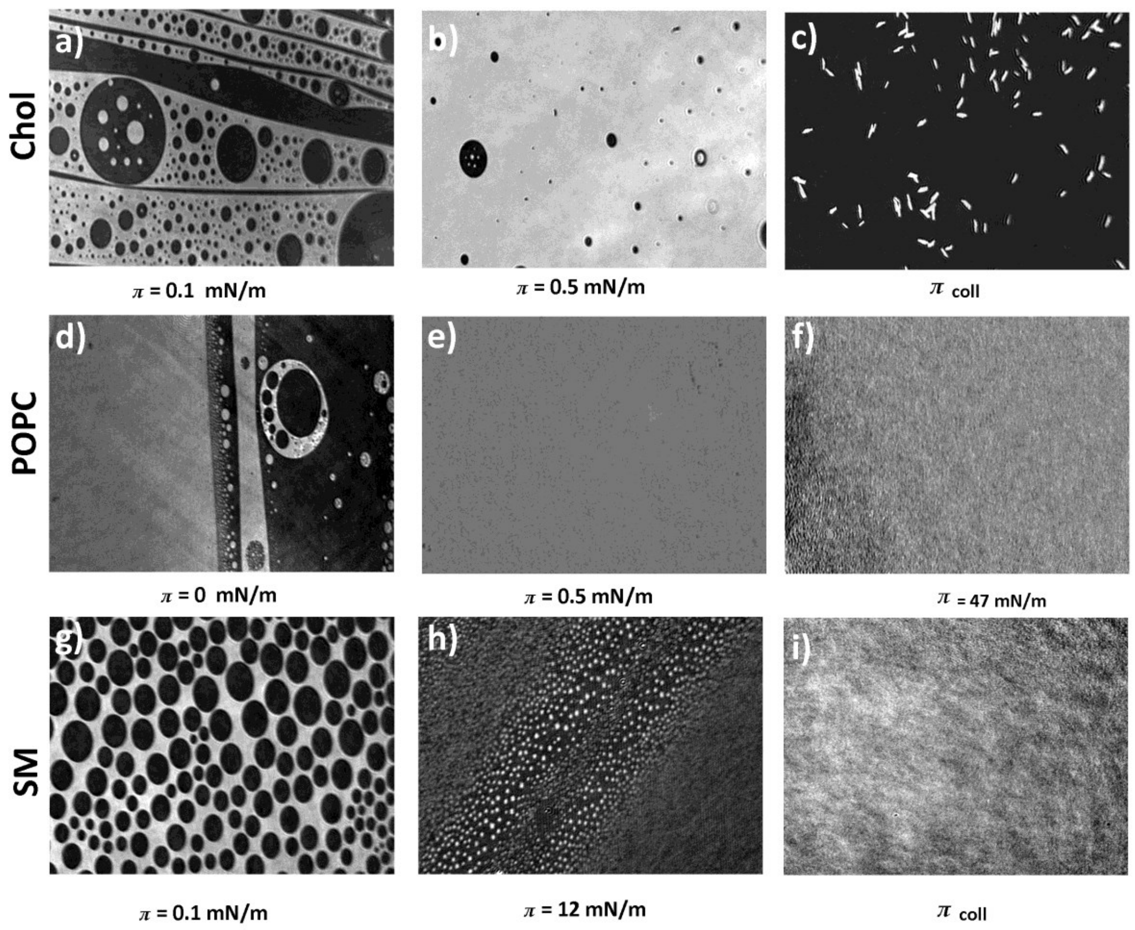

Fig. S2 The texture of pure lipids monolayers: (a-c) cholesterol, (d-f) POPC and (g-i) SM
