# Supplementary material for: Cyclosporin A in Membrane Lipids Environment: Implications for Antimalarial Activity of the Drug—The Langmuir Monolayer Studies
Source: J Membr Biol. 2015 Jun 16;248(6):1021–32. doi: 10.1007/s00232-015-9814-9 (PMC4611017; doi:10.1007/s00232-015-9814-9)
Supplement: Supplementary file 3 — Supplementary material 3 (PDF 476 kb) [file 232_2015_9814_MOESM3_ESM.pdf]

Supplementary Material 3

The Journal of Membrane Biology

Cyclosporin A in Membrane Lipids Environment – Implications for Antimalarial Activity of the Drug. The Langmuir Monolayer Studies

Patrycja Dynarowicz-Łątka*, Anita Wnętrzak, Katarzyna Makyła-Juzak

*Corresponding author: ucdynaro@cyf-kr.edu.pl

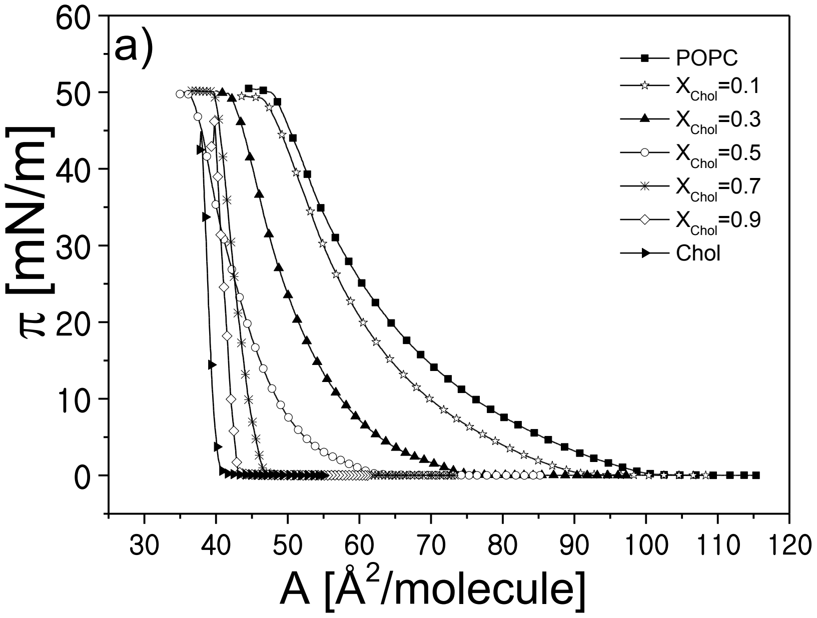

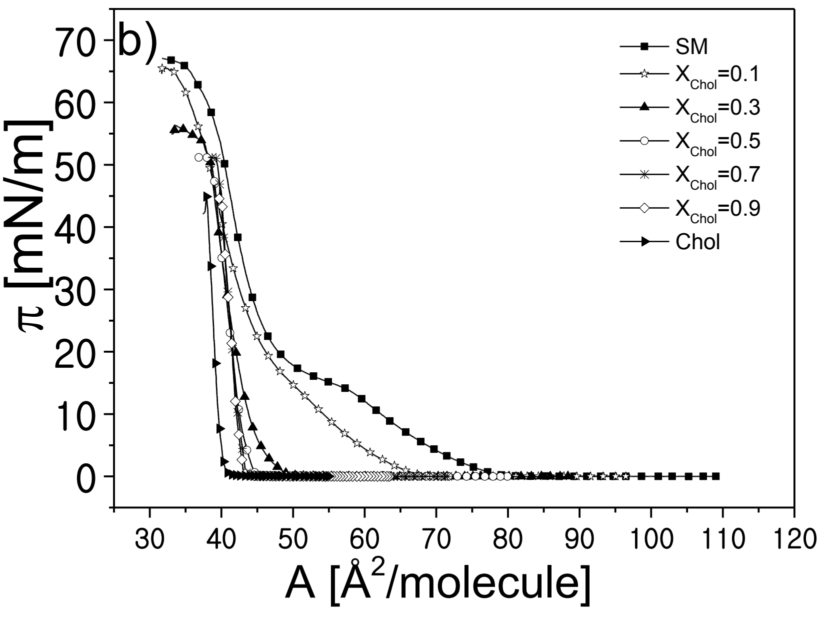

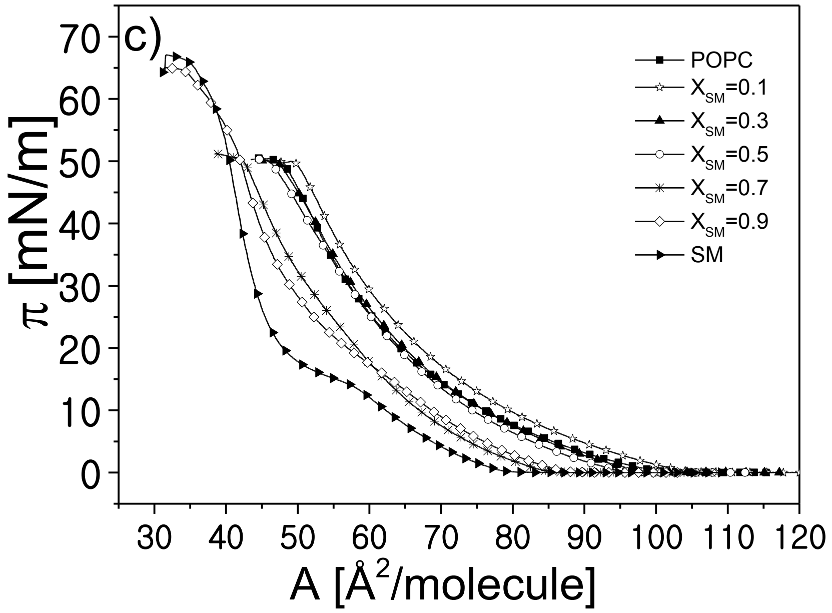

Fig. S3 Surface pressure (π) - area (A) isotherms of mixed systems: (a) POPC/Chol; (b) SM/Chol and (c) POPC/SM
