# Supplementary material for: Cyclosporin A in Membrane Lipids Environment: Implications for Antimalarial Activity of the Drug—The Langmuir Monolayer Studies
Source: J Membr Biol. 2015 Jun 16;248(6):1021–32. doi: 10.1007/s00232-015-9814-9 (PMC4611017; doi:10.1007/s00232-015-9814-9)
Supplement: Supplementary file 4 — Supplementary material 4 (PDF 369 kb) [file 232_2015_9814_MOESM4_ESM.pdf]

Supplementary Material 4

The Journal of Membrane Biology

Cyclosporin A in Membrane Lipids Environment – Implications for Antimalarial Activity of the Drug. The Langmuir Monolayer Studies

Patrycja Dynarowicz-Łątka*, Anita Wnętrzak, Katarzyna Makyła-Juzak

*Corresponding author: ucdynaro@cyf-kr.edu.pl

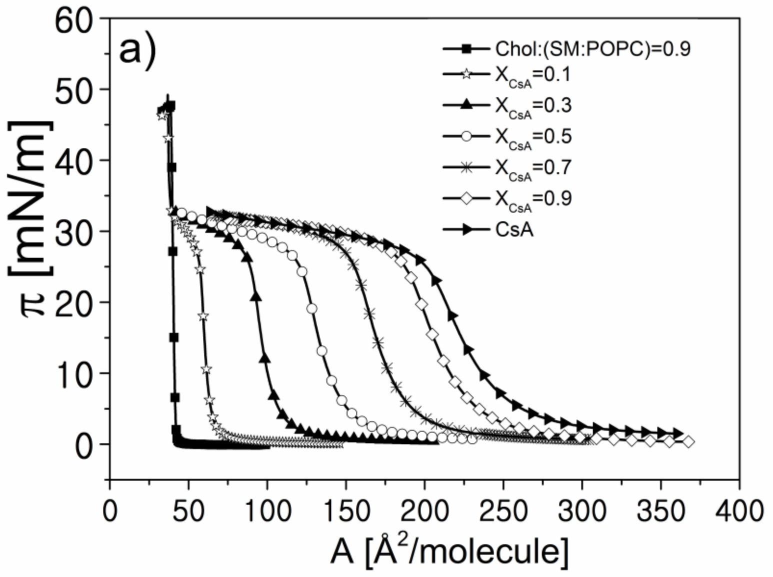

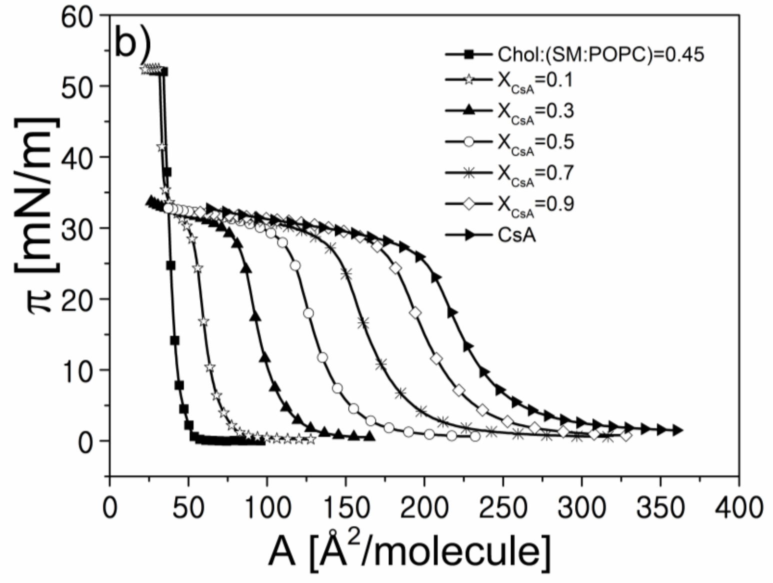

Fig. S4 Surface pressure (π) - area (A) isotherms for model normal (a) and infected membrane (b) and their mixtures.
